# Prevalence and risk factors of incidental prostate cancer in certain surgeries for benign prostatic hyperplasia: A systematic review and meta-analysis

**DOI:** 10.1590/S1677-5538.IBJU.2021.0653

**Published:** 2022-11-01

**Authors:** Zhenlang Guo, Junwei He, Lijuan Huang, Zhaohui Wang, Ping Hu, Shusheng Wang, Zunguang Bai, Jun Pan

**Affiliations:** 1 The Second Affiliated Hospital of Guangzhou University of Chinese Medicine Department of Urology Guangzhou China Department of Urology, The Second Affiliated Hospital of Guangzhou University of Chinese Medicine, Guangzhou, China; 2 The First Affiliated Hospital of Sun Yat-Sen University Department of Organ Transplant Guangzhou China Department of Organ Transplant, The First Affiliated Hospital of Sun Yat-Sen University, Guangzhou, China

**Keywords:** Prostatic Hyperplasia, Meta-Analysis as Topic, Prevalence, Risk Factors

## Abstract

**Background:**

This study aimed to explore the prevalence and clinical risk factors in patients diagnosed with incidental prostate cancer (IPC) during certain surgeries (transurethral resection of the prostate [TURP], open prostatectomy [OP], and holmium laser enucleation of the prostate [HoLEP]) after clinically suspected benign prostatic hyperplasia (BPH).

**Materials and Methods:**

Literature search of the MEDILINE, Web of Science, Embase, and Cochrane Library databases was performed to identify eligible studies published before June 2021. Multivariate adjusted odds ratios (ORs) and associated 95% confidence intervals (CIs) of the prevalence and clinical risk factors of IPC were calculated using random or fixed-effect models.

**Results:**

Twenty-three studies were included in the meta-analysis. Amongst the 94.783 patients, IPC was detected in 24.715 (26.1%). Results showed that the chance of IPC detection (10%, 95% CI: 0.07-4.00; P<0.001; I2=97%) in patients treated with TURP is similar to that of patients treated with HoLEP (9%, 95% CI: 0.07-0.11; P<0.001; I2=81.4%). However, the pooled prevalence estimate of patients treated with OP was 11% (95% CI: −0.03-0.25; P=0.113; I2=99.1%) with no statistical significance. We observed increased incidence of IPC diagnosis after BPH surgery amongst patients with higher prostate-specific antigen (PSA) level (OR: 1.13, 95% CI: 1.04-1.23; P=0.004; I2=89%), whereas no effect of age (OR: 1.02, 95% CI: 0.97-1.06; P=0.48; I2=78.8%) and prostate volume (OR: 0.99, 95% CI: 0.96-1.03; P=0.686; I2=80.5%) were observed.

**Conclusions:**

The prevalence of IPC was similar amongst patients undergoing TURP, HoLEP, and OP for presumed BPH. Interestingly, increased PSA level was the only independent predictor of increasing risk of IPC after BPH surgery rather than age and prostate volume. Hence, future research should focus on predictors which accurately foretell the progression of prostate cancer to determine the optimal treatment for managing patients with IPC after BPH surgery.

## INTRODUCTION

Prostate cancer is the most common malignant tumor amongst ageing male patients, with an estimated 41.000 Americans dying from prostate cancer annually (1). Incidental prostate cancer (IPC) is defined as a tumor incidentally diagnosed after surgery for benign prostatic hyperplasia (BPH) (prostate cancer was not suspected before) or found after autopsy or incidentally detected after radical cystoprostatectomy for patients with bladder cancer (2–4). Moreover, patients with BPH are usually screened for prostate cancer before surgery to rule out the presence of IPC that may ultimately change the treatment strategy (5). Thus, previous studies have revealed that IPC detection after BPH surgery has declined in the prostate-specific antigen (PSA) era (4, 6). Some studies have shown that the prevalence of IPC between patients diagnosed with BPH undergoing TURP is between 5% and 14% (7, 8). A recent study has indicated that the clinical course of IPC has become aggressive, although most IPCs are not clinically obvious (9). The decision ‘treatment or not’ should be determine by predictive factors that can accurately foretell IPC after BPH surgery (10). However, the best clinical management of IPC has remained controversial for decades.

In the present study, we conducted a systematic review and meta-analysis of previous literature to explore the prevalence and clinical risk factors in patients diagnosed with IPC for surgery after clinically suspected BPH. We also performed subgroup and meta-regression analyses to determine how the potential variables affected the merged results and the level of heterogeneity of the meta-analysis.

## MATERIALS AND METHODS

The methods of this meta-analysis were conducted in accordance with the Cochrane Collaboration criterion (11). The Preferred Reporting Items for Systematic Reviews and Meta-Analyses (PRISMA) statement was utilized for reporting our study (Supplementary Material 1) (12). Thus, no ethical approval and patient consent were required.

### Search strategy

Literature search of the MEDILINE, Web of Science, Embase, and Cochrane Library databases were performed to identify eligible studies on the prevalence and clinical risk factors in patients diagnosed with IPC during surgery after clinically suspected BPH published before June 2021. Each database was searched without restrictions in language, publication type, or region by using the following combination of Medical Subject Headings (MeSH) and non-MeSH search terms: (‘prostate cancer’ OR ‘prostate neoplasm’) AND (‘incidental’) AND (‘benign prostatic hyperplasia’). Moreover, a freehand search was conducted for additional relevant articles of interest in journals not listed in these databases. Any discrepancy was settled by consulting amongst investigators.

### Inclusion and exclusion criteria

All publications regarding the prevalence and risk factors in patients diagnosed with IPC after BPH surgery were included if they met the following eligibility criteria: (1) original studies regarding the relevant topic; (2) the primary endpoint was IPC prevalence and/or associated risk factors amongst IPC patients (as previously defined) after BPH surgery (transurethral resection of the prostate [TURP], open prostatectomy [OP], and holmium laser enucleation of the prostate [HoLEP]); and (3) studies reporting sufficient data of prevalence and risk estimates with corresponding 95% confidence intervals (CIs) or sufficient data to calculate them. The exclusion criteria were as follows: (1) non-English articles; (2) case reports, editorial comments, and review articles; and (3) patients have been diagnosed with prostate cancer preoperatively. Furthermore, repeat publications from the same authors or the same centre were excluded to avoid duplication of information, and we retained only the most recent or largest study (where appropriate). Disagreements were resolved through discussion amongst investigators.

### Data extraction and methodological-quality assessment

Data extraction was conducted by two independent investigators using a pre-established data extraction form, and another investigator checked the correctness of all extractions. Any disagreement was resolved by the adjudicating senior authors. The following data were extracted using a standardized Excel (Microsoft Corporation) file: first author, publication year, country, database source and duration, study design, participants characteristics (i.e., mean age, mean prostate volume, mean PSA, sample size, and number of patients diagnosed with IPC), prevalence, and risk estimates with their corresponding 95% CIs or sufficient original data. Moreover, if potentially eligible records did not provide sufficient information, we contacted the primary authors to acquire missing data.

The quality of the included studies was assessed by two independent reviewers according to the Newcastle-Ottawa scale (NOS) (13), which comprises nine items. Each item was evaluated as either ‘yes’, ‘no’, or ‘unclear’, which corresponded to ‘1’, ‘0’, or ‘0’ in accordance with the information provided by the studies. The total score ranged from 0 to 9 and was categorized as follows: a score of 8 to 9 was considered high quality, a score of 6 to 7 was considered moderate quality, and a score of 5 or below was considered low quality. Disagreements were resolved by discussion amongst investigators.

### Data synthesis and analysis

The overall prevalence of IPC and risk estimates of the predictors were calculated through the prevalence and odds ratios (ORs) with their corresponding 95% CIs by using STATA software (version 15.0; serial number: 10699393; StataCorp Wyb). The I-square (I2) test was applied to evaluate the study heterogeneity, with I2 values of 0%, 25%, 50%, and 75% representing no, low, moderate, and high heterogeneity, respectively. Heterogeneity amongst the studies was evaluated by random or fixed-effect models, and a considered severe heterogeneity of I2≥50% was evaluated by random-effect models. Otherwise, a fixed-effect model was used. Statistical significance was set at P <0.05 through two-sample t-test. To explore the influence of various clinical characteristics on heterogeneity, we performed subgroup analyses stratified by different geographic distributions. Sensitivity analysis was conducted by omitting each study individually to assess the quality and consistency of the results. To investigate the possible sources of heterogeneity, a meta-regression analysis was conducted and the restricted maximum likelihood method was applied for analysis. Finally, the test of Egger et al. (14) and Begg & Mazumdar (15) were performed to assess publication bias, and the funnel-plot symmetry was examined.

## RESULTS

Study identification and selection

**Figure 1:**
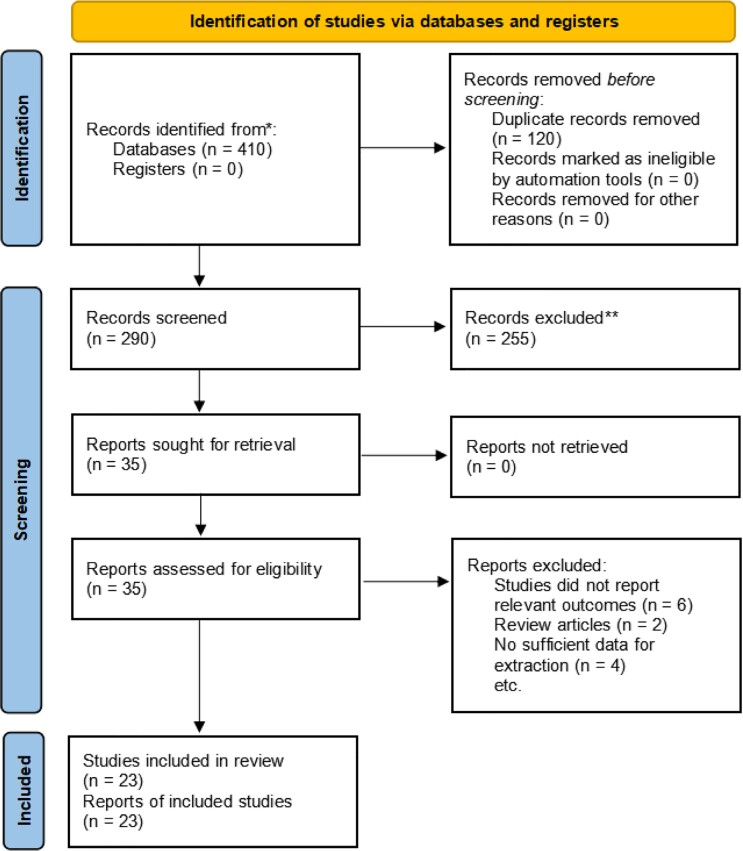
Flow diagram of literature searches according to the preferred reporting items for systematic reviews and meta-analyses statement.

A total of 410 records were identified initially based on the comprehensive search strategy described at the search stage. Figure-1 presents the process of study selection. After removing 120 duplicates, only 290 unique articles remained. Then, 35 articles were further assessed through their full texts after screening the titles and abstracts of 290 articles in detail. However, 12 articles were excluded for the following reasons: 6 studies did not report relevant outcomes, 2 studies were reviews, and 4 studies had insufficient data for extraction. Amongst them, 23 observational studies (8, 16–37) comprising 94.783 patients with 24.715 (26.1%) IPC were included in the meta-analysis in accordance with the eligibility criteria.

### Study characteristics and methodological-quality assessment

Overall, the basic characteristics of the included studies are illustrated in Table-1. The included studies (21) were retrospective studies (8, 16, 18–30, 32–37), 1 was a prospective study (31), and 1 was a cross-sectional study (17) that were published between 2005 and 2021, with sample sizes ranging from 120 patients to 76.788 patients. Furthermore, mean age ranged from 66 years to 75.8 years, mean prostate volume ranged from 44.2mL to 110mL, and mean PSA level ranged from 2.9ng/mL to 21.47ng/mL. Amongst the included studies, twelve were performed in Europe (17–19, 21, 23, 25, 26, 32–34, 36, 37), six in Asia (8, 16, 24, 28, 29, 35), two in Africa (20, 22), two in North America (27, 30), one in Oceania (31). Importantly, 18 studies reported clinical stage of IPC (T1a and T1b) (8, 18–23, 25–27, 30–37). All studies were published in English. Finally, three surgical methods (TURP, OP, and HoLEP) for BPH were reported amongst all included studies.

**Table 1:** Characteristics of the included studies.

First author year	Study design	Country	Database source (Duration)	Sample size (No. IPC)	Stage	Mean age, years	Mean PV, mL	Mean PSA, ng/mL	Risk factors	Prevalence, %
Abedi AR. 2018 (16)	Retrospective study	Iran	Shohada-e-Tajrish Hospital database (2006-2016)	TURP:315 OP:108 (TURP:40, OP:44)	NA	68.74±9.87	80.56±25.12	21.47±13.44	NA	TURP:12.6 OP:40.7
Andrèn O. 2009 (17)	Cross-sectional study	Sweden	Swedish National Inpatient Register (1970-2003)	TURP:72322 OP:4456 (23.288)	NA	NA	NA	NA	NA	OP:30.3
Argyropoulos A. 2005 (18)	Retrospective study	Greece	Athens General Hospital (1999-2003)	TURP:786 (34)	T1a:17 T1b:17	69.7	NA	5.1	NA	TURP:4.3
Capogrosso P. 2018 (19)	Retrospective study	Italy	European academic center (2007-2016)	OP:139 TURP:498 HoLEP:540 (74)	T1a:64 T1b:10	66	76	3.2	PV, age and PSA	OP:6.4
Elkoushy MA. 2015 (20)	Retrospective study	Egypt	HoLEP database (1998-2014)	HoLEP:1242 (70)	T1a:54 T1b:16	75.8±8.7	NA	NA	Age and PSA	HoLEP:5.64
Froehner M. 2009 (21)	Retrospective study	Germany	NA (1997-2006)	TURP:693 (70)	T1a:52 T1b:18	NA	NA	NA	NA	TURP: 10.1
Gunda D. 2018 (22)	Retrospective study	Tanzaia	Bugando University Hspital in Tanzania (2015)	TURP:152 (33)	T1a:11 T1b:22	69±9.4	92.7	8.5	PV, age and PSA	TURP:21.71
Herlemann A. 2017 (23)	Retrospective study	Germany	Department of Urology of the Ludwig-Maximilians-University of Munich (2013-2014)	TURP:229 HoLEP:289 (TURP:39, HoLEP:43)	T1a:14 T1b:68	71	80	5.5	Age and PSA	TURP:17 HoLEP:15
Kim M. 2014 (24)	Retrospective study	Korea	Seoul National University College of Medicine (2008-2011)	HoLEP:458 (27)	NA	68.4±6.6	NA	3.38±4.16	Age and PSA	HoLEP:5.9
Matanhelia DM. 2019 (25)	Retrospective study	Ireland	Mater Misericordiae University Hospital (2007-2016)	TURP:826 (72)	T1a:37 T1b:35	73.3	44.2	5.25	NA	TURP:8.7
Misraï V. 2019 (26)	Retrospective study	France	Rennes Hospital (2013-2018)	OP:393 HoLEP:345 (OP:33, HoLEP:34)	T1a:28 T1b:39	69	110	6.6	NA	OP:8.5 HoLEP:9.9
Nunez R. 2011 (27)	Retrospective study	America	Department of Urology of Mayo Clinic (2007-2010)	HoLEP:240 (28)	T1a:14 T1b:14	73	71.2	3.3	NA	HoLEP:11.7
Ohwaki K. 2017 (28)	Retrospective study	Japan	St. Luke's International Hospital (2008-2014)	HoLEP:654 (41)	NA	70	66	6.1	PV and PSA	HoLEP:6.3
Otsubo S. 2015 (29)	Retrospective study	Japan	Southwest Urological Clinic of Japan (2006-2011)	HoLEP:365 (25)	NA	68	55.5	4.5	NA	HoLEP:6.8
Otto B. 2014 (30)	Retrospective study	America	New York-Presbyterian Hospital (2006-2011)	TURP:760 (11)	T1a:9 T1b:2	71	92.4	NA	NA	TURP:1.4
Perera M. 2016 (31)	Prospective study	Australia	Ludwig Institute for Cancer Research, Austin Hospital (2010-2013)	TURP:923 (243)	T1a:109 T1b:134	NA	65	NA	NA	TURP:26.3
Pirša M. 2018 (32)	Retrospective study	Croatia	Department of Urology in Sestre milosrdnice University Hospital Center (1997-2017)	TURP:4.372 (265)	T1a:119 T1b:146	74.5	56	NA	NA	TURP:6.1
Porcaro AB. 2021 (33)	Retrospective study	Italy	Department of Urology of University of Verona (2017-2019)	TURP:389 (18)	T1a:11 T1b:7	70	55	2.9	NA	TURP:4.6
Rosenhammer B. 2018 (34)	Retrospective study	Germany	University of Regensburg (2016-2017)	TURP:60 HoLEP:60 (TURP:5, HoLEP:14)	T1a:12 T1b:7	71.5±7.9	74.2±13.9	4.99±3.12	PV, age and PSA	TURP:8.3 HoLEP:23.3
Sakamoto H. 2014 (35)	Retrospective study	Japan	Tokyo Saiseikai Central Hospital (2006-2011)	TURP:307 (31)	T1a:18 T1b:13	69.2	61	5.4	PV, age and PSA	TURP:10.1
Skrzypczyk MA. 2014 (36)	Retrospective study	Poland	Centre of Postgraduate Medical Education in Warsaw (2004-2010)	OP:145 TURP:823 (34)	T1a:19 T1b:15	71	70	3.36	PSA	OP:3.5
Tonyali S. 2021 (37)	Retrospective study	Turkey	Turkiye Yuksek Ihtisas Training and Research Hospital (2008-2018)	OP:36 TURP:281 (21)	T1a:10 T1b:11	69	NA	3.24	NA	OP:6.6
Yoo C. 2012 (8)	Retrospective study	Korea	Yonsei University College of Medicine (2004-2008)	TURP:1.613 (78)	T1a:32 T1b:46	71.1±7.6	59.5±30.5	4.7±4.2	PV, age and PSA	TURP:6.6

In general, the methodological quality of the included studies was evaluated on the basis of the NOS. One study (31) acquired eight points and were considered high quality, twenty one studies (8, 16, 18–30, 32–37) acquired six or seven points and were considered moderate quality, and the remaining study (17) scored three and was considered low quality (Supplementary Material 2).

### Prevalence of IPC after BPH surgery

**Figures 2A-2C:**
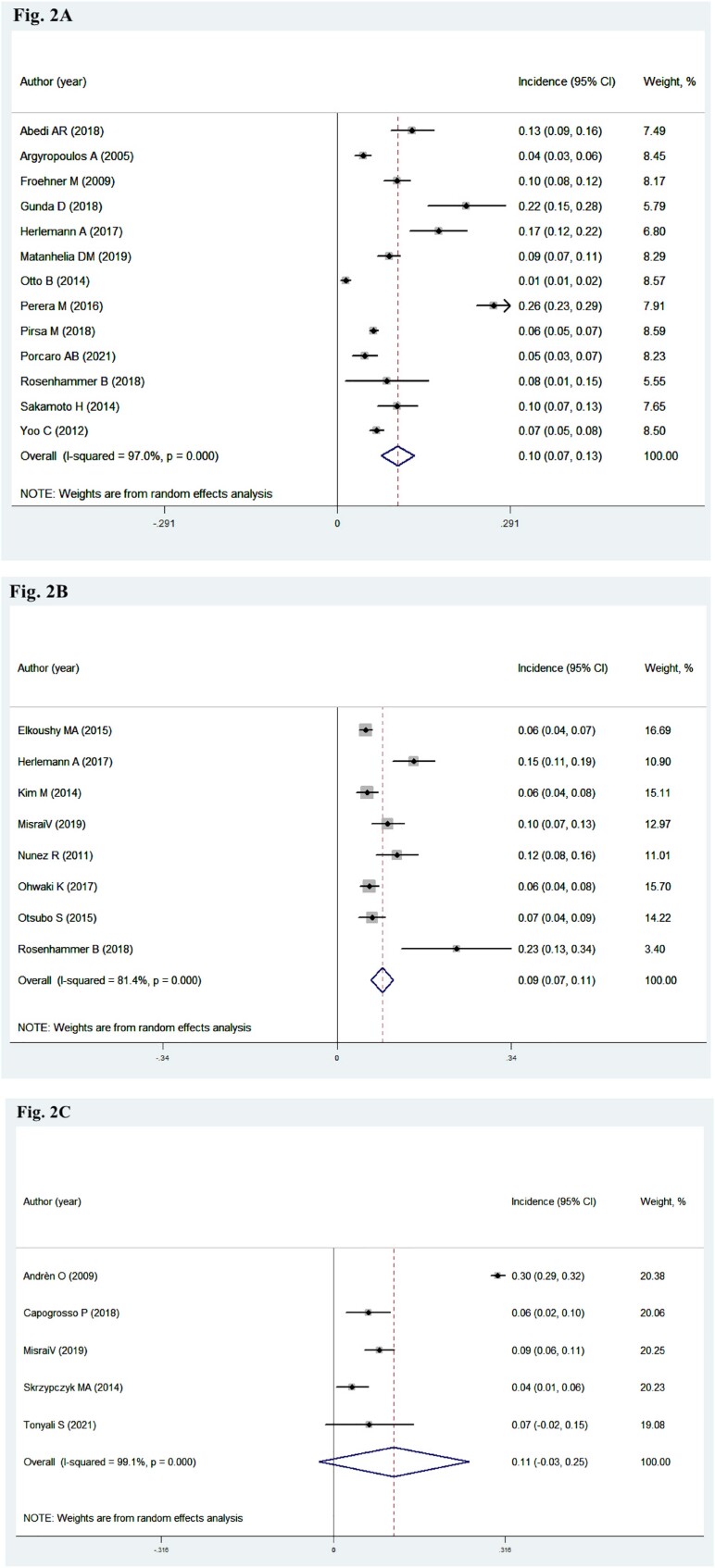
Meta-analysis on the prevalence of IPC after BPH surgery. CI, confidence interval. 2A: TURP group; 2B: HoLEP group; 2C: OP group.

All included studies reported sufficient data on IPC prevalence (8, 16–37). Overall, patients diagnosed with IPC after BPH surgery was detected in 24,715 of 94.783 patients. Results showed that the chance of IPC detection (10%, 95% CI: 0.07-4.00; P <0.001; I2=97%) in patients treated with TURP is similar to that of patients treated with HoLEP (9%, 95% CI: 0.07-0.11; P <0.001; I2=81.4%). However, the pooled prevalence estimate of patients treated with OP was 11% (95% CI: −0.03-0.25; P=0.113; I2=99.1%) with no statistical significance. However, significant heterogeneity was observed, so a random-effect model was applied for pooled analysis (Figures 2A–2C).

In the subgroup analysis stratified by different geographic distributions in patients with IPC after TURP, a higher prevalence was observed in Oceania (26%, 95% CI: 0.23-0.29; P <0.001) and Africa (22%, 95% CI: 0.15-0.28; P <0.001), followed by Asia (9%, 95% CI: 0.06-0.13; P <0.001), Europe (8%, 95% CI: 0.06-0.10; P <0.001), and North America (1%, 95% CI: 0.01-0.02; P=0.001). We also conducted a meta-regression analysis to further investigate the possible sources of heterogeneity, and results revealed that none of the covariate (geographic distribution, P=0.958) resulted in heterogeneity amongst the included studies. Moreover, sensitivity analysis demonstrated that the stability of the results exhibited no significant change by omitting each study individually (Table-2). Finally, potential publication bias was likely to exist according to inspection of formal statistical tests (Begg test, P=0.044; Egger test, P=0.022).

**Table 2:** Results of sensitivity analyses.

Study omitted	OR	95% CI	
Abedi AR (2018) (16)	0.1	0.07	0.13
Argyropoulos A (2005) (18)	0.11	0.1	0.14
Froehner M (2009) (21)	0.1	0.07	0.13
Gunda D (2018) (22)	0.09	0.07	0.12
Herlemann A (2017) (23)	0.1	0.07	0.12
Matanhelia DM (2019) (25)	0.1	0.07	0.13
Otto B (2014) (30)	0.11	0.08	0.14
Perera M (2016) (31)	0.08	0.06	0.11
Pirsa M (2018) (32)	0.11	0.07	0.14
Porcaro AB (2021) (33)	0.11	0.07	0.14
Rosenhammer B (2018) (34)	0.1	0.07	0.13
Sakamoto H (2014) (35)	0.10	0.07	0.13
Yoo C (2012) (8)	0.11	0.07	0.13
Combined	0.1	0.07	0.13

OR = odds ratios; CI = confidence interval

### Risk factors of IPC after BPH surgery

**Figures 3A-3C:**
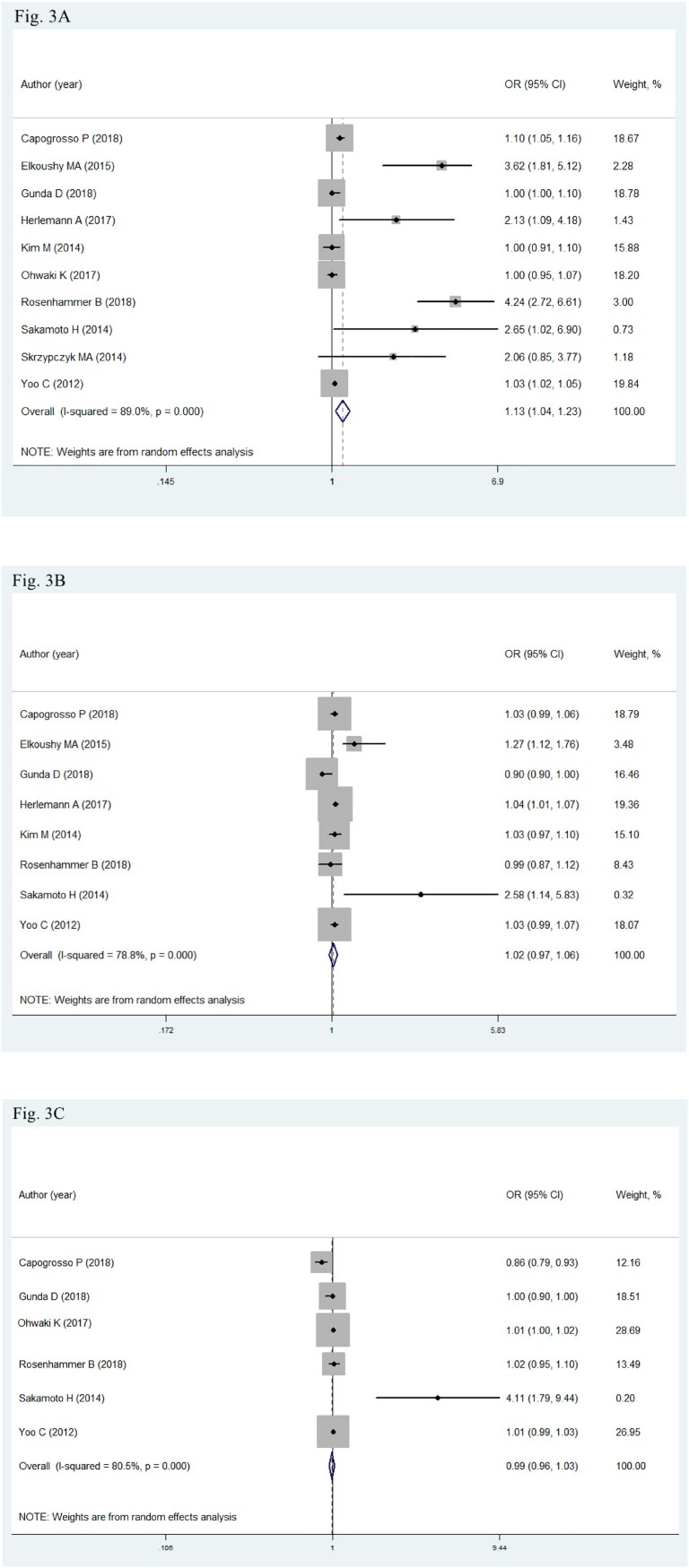
Meta-analysis on the risk factors of IPC after BPH surgery. OR, odds ratios; CI, confidence interval. 3A: PSA; 3B: Age; 3C: Prostate volume.

Nine retrospective cohorts comprising 6.241 patients reported data on risk factors of IPC (8, 19, 20, 22–24, 28, 34, 35). We observed increased incidence of IPC diagnosis after BPH surgery amongst patients with increased PSA level (OR: 1.13, 95% CI: 1.04-1.23; P=0.004; I2=89%) in multivariate analysis. However, no effect of age (OR: 1.02, 95% CI: 0.97-1.06; P=0.48; I2=78.8%) and prostate volume (OR: 0.99, 95% CI: 0.96-1.03; P=0.686; I2=80.5%) were observed possibly due to the limited number of included studies. Moreover, a random-effect model was utilized due to the significant heterogeneity (Figures 3A–3C).

## DISCUSSION

### Main findings

Uncertainty currently exists about the prevalence and risk factors of IPC with conflicting opinions based on single institutional research. Accordingly, we performed this systematic review and meta-analysis to address these issues. Our results revealed that the prevalence of IPC was similar amongst patients undergoing TURP, HoLEP, and OP for presumed BPH. Higher prevalence was also observed in Oceania and Africa, followed by Asia, Europe, and North America. Notably, sensitivity analysis indicated that the stability of the results had no significant change by omitting each study individually, although the subgroup and meta-regression analyses could not identify the potential factors that may affect the level of heterogeneity between studies. However, potential publication bias was likely to exist according to inspection of formal statistical tests. Interestingly, increased PSA level was significantly associated with increased risk of IPC after BPH surgery rather than age and prostate volume.

Although our results showed that PSA was a highly sensitive predictor for IPC detection, it may have a higher rate of false positives because its specificity was not high. Indeed, almost all included studies reported a significant association between PSA and IPC detection, whereas two studies yielded conflicting results (24, 28). Kim et al. (24) retrospectively analyzed 458 consecutive patients who underwent HoLEP. They found that PSA does not affect the detection rate of IPC after HoLEP (OR: 0.999, 95% CI: 0.908-1.098; P=0.976) in multivariate analysis. Additionally, Ohwaki et al. (28) performed a retrospective study comprising a consecutive group of 688 patients who were diagnosed with BPH and underwent HoLEP. They observed no effect of PSA. Interestingly, diabetes may be an important factor for predicting IPC (OR: 3.15, 95% CI: 1.06-9.43; P=0.04) in men diagnosed with BPH who have undergone HoLEP. When we discarded these two studies from the meta-analysis, the results showed no significant changes, thereby validating the rationality and reliability of our analysis.

### Implications for clinical practice

A significant increase has been observed in the number of minimally invasive surgical treatments for BPH, which is considered to be a factor affecting the incidence of IPC detection after BPH surgery (38). In fact, these surgical methods may not provide a sufficient amount of prostate tissue for pathological examinations. We found that the proportion of patients receiving HoLEP treatment significantly increased compared with those receiving TURP and OP. Unexpectedly, patients treated with TURP were more likely to be diagnosed with IPC rather than HoLEP and OP according to our meta-analysis. Therefore, these results should be interpreted rigorously. Generally, the surgical concepts of prostate tissue removal for the three types of BPH surgeries are similar. The previous literature demonstrated that HOLEP have a higher total detection rate of incidental PCa when compared with TURP due to more efficient tissue removal (34), which was inconsistent with our results. However, other previous studies indicated that the probability of IPC detection and the quality of the tissue retrieved after surgery were not significantly different among TURP, HoLEP, or OP (23, 39). One of the reasons may be that some prostatic tissue retrieved by HoLEP is lost due to coagulative and vaporizing effects. Therefore, it is more difficult to find biological reasons to explain the different detection rate of IPC between them. Future research should pay attention to the quality of prostate tissue retrieved through different BPH surgical methods to further determine the reasons for the different prevalence of IPC except for the sample size (39). Moreover, the best treatment for IPC remains controversial. Active surveillance for every patient with IPC subclassified as clinical stage T1a or T1b after BPH surgery is unacceptable. Thus, patients who do not meet the criteria for active surveillance can be recommended for radiotherapy or radical prostatectomy. Radiotherapy is safe for patients with a history of BPH surgery and is related to an acceptable quality of life. However, undergoing radical prostatectomy is technically challenging for patients with a history of TURP (40). Our analysis showed no significant difference in terms of patients age and prostate volume rather than PSA level. However, other potential risk factors such as PSA density, PSA velocity, or underlying diseases could not be investigated due to the limited data obtained from the included studies. Nevertheless, one previous research indicated that higher preoperative PSA density and velocity, preoperative treatment with 5-alpha reductase inhibitors, and diabetes was identified to have a significant correlation with the diagnosis of IPC after surgery for BPH (41–44). Future study regarding more important risk factors of IPC with sufficient data are still needed for early screening and identification of IPC patients. Abedi et al. (18) indicated that the cut-off point of PSA for detecting IPC was 3.8ng/mL, which showed low sensitivity and high specificity. The relevant information regarding this issue was limited reported in all the included studies. Hence, future studies with high-quality should focus on the PSA referral cut-off value for the diagnosis of IPC after BPH surgery, so as to improve the early recognition of IPC.

### Strengths and limitations

Our meta-analysis exhibited crucial strengths in several ways. Firstly, to our knowledge, the present meta-analysis is the first one focusing on the prevalence and clinical risk factors of IPC after BPH surgery. Moreover, sensitive and meta-regression analyses were performed to determine the potential factors that moderated the level of heterogeneity and results of the meta-analysis according to the PRISMA guidelines. Secondly, multivariate-adjusted risk estimates were applied to minimize the other relevant confounding factors that may influence the overall results. Lastly, the results of the sensitivity analysis and meta-regression validated the rationality and reliability of this meta-analysis.

However, some limitations should also be addressed and merit further discussion when interpreting our results. Firstly, significant heterogeneity was observed. Therefore, the introduction of potentially significant heterogeneity was imminent even though meta-regression was conducted. The reason may be that all included studies were observational design with the disadvantages of heterogeneity and variations in terms of histopathological examination. Secondly, we did not evaluate data regarding the cancer clinical stage (TNM system) of patients because few studies have reported related information. Thirdly, potential publication bias is likely to exist, although our data search included a number of databases combined with freehand search. Moreover, related results may be affected with the continuous advancement of surgical techniques. Finally, our understanding of predictors which accurately foretell prostate cancer progression remains insufficient because related data have been investigated inadequately.

## CONCLUSION

The prevalence of IPC was similar amongst patients undergoing TURP, HoLEP, and OP for presumed BPH. Moreover, increased PSA level was an important predictor of the presence of IPC after BPH surgery. However, no effect of age and prostate volume was observed. Therefore, further prospective studies should be conducted in a multicentric population to evaluate other relevant variables that can accurately predict the progression of prostate cancer to determine the optimal treatment for IPC patients after BPH.

Trial and protocol registration: PROSPERO CRD42021268051.

Zunguang Bai 1*, Jun Pan, MD, 1*
